# Hyperspectral measurements of immature *Lucilia sericata* (Meigen) (Diptera: Calliphoridae) raised on different food substrates

**DOI:** 10.1371/journal.pone.0192786

**Published:** 2018-02-13

**Authors:** Jodie A. Warren, T. D. Pulindu Ratnasekera, David A. Campbell, Gail S. Anderson

**Affiliations:** 1 School of Criminology, Simon Fraser University, Burnaby, BC, Canada; 2 Department of Statistics and Actuarial Science, Simon Fraser University, Burnaby, BC, Canada; Rosalind Franklin University of Medicine and Science, UNITED STATES

## Abstract

Immature *Lucilia sericata* (Meigen) raised on beef liver, beef heart, pork liver and pork heart at a mean temperature of 20.6°C took a minimum of 20 days to complete development. Minimum development time differences within stages were observed between the meat types (pork/beef), but not the organ types (liver/heart). Daily hyperspectral measurements were conducted and a functional regression was completed to examine the main effects of meat and organ type on daily spectral measurements. The model examined post feeding larval spectral measurements of insects raised on beef liver alone, the effect of those raised on pork compared with those raised on beef, the effect of those raised on heart compared with those raised on liver and the interactional effect of those raised on pork heart compared with those raised on beef liver. The analyses indicated that the spectral measurements of post feeding *L*. *sericata* raised on pork and beef organs (liver and heart) are affected by the meat and organ type.

## Introduction

Blow flies (Diptera: Calliphoridae) are holometabolous insects and, in some species, most of the immature stages are associated with carrion or animal remains. These remains are an ephemeral resource that the larval stages depend on for their nutritional requirements. The immature blow fly develops at a predictable rate which is temperature dependent on the known food source and it is precisely that on which medico-legal entomology is based [1].

One area of medico-legal entomology, a subdiscipline offorensic entomology includes examining immature blow fly development in order to estimate the tenure of blow flies developing on decomposing corpses in death investigations. The estimated tenure or time since colonization can then be used to infer the minimum elapsed time since death occurred [2–4].

Unfortunately due to many variables, the minimum time since death, although accurate, is a modest estimate [5]. Since methods of extrapolating time since death are estimates, precision is lessened in the latter stages of the lifecycle as those stages are much lengthier than earlier stages [5]. Finding techniques with more precision in estimating the minimum time since death is of great importance. Forensic entomologists have sought ways to improve current estimation methods by using Gas Chromatography/Mass Spectrometry to identify changes in cuticular hydrocarbons commensurate with development [6–10], Micro-Computed Tomography scanning to assist in identifying morphological changes in intra-puparial development throughout metamorphosis [11], integrating gene expression variations with conventional methods [12–14] and, most recently, examining microorganisms associated with carrion [10, 15–18], all of which improve current methods. Hyperspectral remote sensing has joined the ranks of these methods by improving current means and also contributing to much needed methods offering confidence intervals [5, 19, 20] as gene expression has [12, 21, 22], thereby satisfying the United States National Research Council’s criticisms of many forensic sciences [2, 4, 23, 24].

Hyperspectral remote sensing is a non-intrusive means of sensing and recording reflected energy from a target surface [25]. The use of hyperspectral remote sensing in medico-legal entomology provides a non-destructive technique to record reflectance from the ageing insect and can be used to identify differences in the insect surface over time which can be used further to identify an estimated time within stage of development [19, 20]. Depending on the wavelengths examined, it combines exhaustive details from the visible spectrum, short wave, near and far infrared. In this case, with each measurement, it is a means to identify changes in the target surface of the immature blow fly as it develops. A spectral signature for each day for each target surface is identified and these signatures change daily [19]. These target surface changes can be used to further identify demarcations within immature stages and allow for more precision of time within larval stadia estimations [19, 20] and the intrapuparial period [19, 26].

An obstacle that potentially arises when using laboratory collected data in case analyses is that most laboratory experiments are not completed on entire remains but instead on beef liver or other animal tissues. Differences in development times have been found among different food substrates for several different blow fly species [27–35]. *Protophormia terraenovae* (Robineau- Desvoidy) development on beef liver was shown to be representative of a whole animal (wounded rat carcass), which supports the use of beef liver in laboratory experiments [34]. Comparisons of other species of blow fly development on whole animals to animal tissue has not been done, and so cannot be commented on; only comparisons between tissues have been made [27–33, 35–37]. The effects of the food substrate on hyperspectral measurements have not been examined. The objective of this research was to examine the effects of different food substrates on developing immature *Lucilia sericata* (Meigen) and, consequently, the effects on the hyperspectral measurements of the lengthy post feeding stage.

## Materials and methods

### Insect rearing

Black film canisters positioned on their sides with approximately 50 g of beef liver within were used to collect eggs from two separate colonies of *L*. *sericata* [19]. The two colonies originated from recently wild-trapped flies collected from Burnaby, Langley, and Vancouver, British Columbia and were used within a year of trapping and were provided by Simon Fraser University’s Biological Sciences Department. The colonies were maintained on a diet of water, sugar and milk powder *ad libitum*. Also, beef liver was added to the cages regularly as an oviposition medium.

Once eggs were oviposited (~two hours), they were divided among 16 treatments, four each of beef liver, beef heart, pork liver and pork heart. Eggs were divided such that each treatment received an estimated 240 eggs combined from the two colonies. Each treatment consisted of a one gallon/4L wide mouth glass jar with approximately a five centimetre depth of moistened sawdust topped by a folded industrial paper towel and the appropriate meat source (approximately 250g). An estimated 200–240 combined eggs from the colonies were placed onto each meat type in each treatment. The number of eggs were estimated based on egg mass size. Each jar was secured with two pieces of industrial paper towel and two elastic bands to prevent escape during the post feeding stage.

All treatments were placed into a Conviron^®^ E/7 environmental chamber set for 75% relative humidity and a 14:10 (L:D) photoperiod. A mean constant temperature of 20.6°C was maintained in the chamber and recorded by ACR Systems Inc. Smartbutton^®^ data loggers and confirmed daily with Fisherbrand^™^ thermometers. The treatments were rotated daily to account for temperature differences within the chamber. Development stage reached was recorded daily and was presented as thermal units, accumulated degree days (ADD). A base temperature of 0°C was applied since the base temperature for this species is unknown for this geographic location [38]. To calculate, [39]

$$ADD={Time}_{\left(days\right)}\;X\;({Temperature}_{\left({}^{\circ}C\right)}-lower\;{threshold}_{\left({}^{\circ}C\right)}).$$

### Spectral measuring

Spectral measurements of ten post feeding *L*. *sericata* from each of the 16 treatments were taken using an ASD (Analytical Spectral Devices^™^, Boulder CO) LabSpec 4 Bench Benchtop Analyzer Spectrometer. All measured larvae were washed with deionized water and patted with filter paper and finally patted with dry filter paper to dry them before measuring. Following the measurements, the larvae were placed back into the treatment container. Measurements were completed in a blackened laboratory to ensure that measurements were of the larvae and not interfering reflecting surfaces. All surfaces and instruments were painted with a matte black paint and the light in the room was that of the light source only. The minimal light from the turned away computer screen and from under the door were consistent and trivial to the measurements.

Each treatment was removed from the environmental chamber once daily beginning at noon and 10 insects were measured from each treatment. Point measurements were taken from the anterior, middle and posterior regions of the washed insect. Calibration using a Spectralon^™^ panel was completed before starting and following every five to seven measurements. A Spectralon^™^ panel is a pure diffuse reflectance standard and is the baseline against which all measurements were compared. A black reference was completed each time with the process of optimization of the spectrometer.

Data files were collected by RS^3™^ software, the program that is specific to ASD spectrometers. Viewspec pro^™^ was then used to convert the files to text files. Mathworks^™^ Matlab formulae were then used to transfer the files and organize the Matlab files by day, meat type and region of measurement to be manipulated for statistical analyses along with fdaM (functional data analysis Matlab) tools (http://www.psych.mcgill.ca/misc/fda/downloads/FDAfuns/).

### Functional model

The raw spectral reflectance observations, X_i_(w) across wavelength (w) for insect i on day Y*i* were smoothed using a 6^th^ order B-Spline basis while controlling roughness through a 3^rd^ derivative penalty to reduce the noise associated with the raw spectra [40]. Smoothing was performed using generalized cross validation to ensure the resulting smooth functions X__i,smooth_(w) tracked the signal without succumbing to the minute level of noise in the reflectance data.

The functional data approach avoids subjectively binning the reflectance across intervals of wavelength and instead treats an entire reflectance curve as a single functional observation. The functional data analysis approach assumes that reflectance varies smoothly with changes in wavelength which coincides with the spectral leakage exhibited by frequency domain correlation. The maximum spectral reflectance scale for each observation was set to one and data were also scaled to have an average reflectance value of zero between 400 and 550 nm wavelengths.

The functional regression equations, where *β(w)* is the contributing regression coefficient for spectral measurements from insects raised on beef liver alone, is given below:

$$Y_{i}=\int_{350}^{2500}X_{i,smooth}(w)\beta (w)dw\;\ldots$$ (1A)

$$+\int_{350}^{2500}X_{i,smooth}(w)\beta_{Pork}(w)dw$$ (1B)

$$\ldots \;+\int_{350}^{2500}X_{i,smooth}(w)\beta_{Heart}(w)dw$$ (1C)

$$\ldots \;+\int_{350}^{2500}X_{i,smooth}{(w)\beta }_{Pork\;Heart}(w)dw,$$ (1D)

The coefficient function *β*_*Pork*_*(w)* allows for differences in the spectral measurement on the day of development due to changing from beef to pork measurements regardless of organ. The coefficient function *β*_*Heart*_*(w)* allows for differences in the spectral measurement on the day of development due to changing from liver to heart measurements regardless of meat type. The coefficient function *β*_*Pork Heart*_*(w)* allows for an interaction in the differences in the spectral measurement on the day of development due to changing from beef liver to pork heart. The model (1a-1d) has additive coefficients from a beef liver baseline. As such, predicting day Y_i_ with spectral reflectance X(w) for a pork liver substrate, the model terms used are 1a and 1b. Predicting the day from a beef heart substrate uses 1a and 1c, and predictions when a pork heart substrate is used, all terms 1a-d are applied.

The goal was to predict the development day based on the spectral reflectance curves and to see if the reflectance is affected by changes in meat type and organs used for rearing the insects. A test for the interaction effect of changing from beef liver to pork heart was performed through testing the null hypothesis that *β*_*Pork Heart*_*(w) = 0* for all wavelengths, w. Regardless of whether or not there was an interaction effect then the main effect of moving from beef to pork irrespective of organ type can be tested with the null hypothesis that *β*_*Pork*_*(w) = 0* for all wavelengths. Similarly, the main effect of moving from liver to heart can be tested with the null hypothesis *β*_*Heart*_*(w) = 0* for all wavelengths. Finally, a test of significance of the reflectance in estimating the day of development can be performed by testing the null hypothesis that *β(w) = 0* for all w.

All of the functional coefficients *β(w)* were modelled as 6^th^ order B-Spline functions with a roughness penalty on their 3^rd^ derivative to prevent unrealistic fluctuations in the reflectance effect across nearby wavelength. The roughness penalties for the X(w) and *β(w)* were determined via cross validation so as to avoid overfitting. The model in (1a-1d) is not a regression model with 2500 covariates per observations but instead the smooth functional form of the reflectance curve is exploited to transform the reflectance measurements into a single functional covariate that happens to span 2500 wavelengths. The estimated coefficient functions *β(w)*, highlight smoothly varying regions of the 800 reflectance curves for each of the three body regions measured that assist in estimating the day of development. All of the estimation uncertainty, from the initial reflectance smoothing to the estimation of the coefficient functions *β(w)*, is carried forward into producing confidence intervals and inference.

## Results

*Lucilia sericata* raised at an average of 20.6°C took a minimum of 20 days to complete immature development on beef liver, beef heart, pork liver and pork heart. Development stage reached and accumulated degree days (ADD) with 0°C base temperature is presented for each of the meat substrates in Table 1. An extra day was spent in the feeding third instar on each of the pork substrates compared with the beef substrates but development to the adult stage took the same number of days. The insects raised on pork were in the intra-puparial period for nine days rather than 10. Although not measured, based on observation alone, the feeding larvae were smaller on the pork substrates compared with the beef substrates but caught up in size to those feeding on the beef substrates with the extra day of feeding.

**Table 1:** The minimum development stage and accumulated degree days (ADD) of *Lucilia sericata* raised at a mean constant temperature of 20.6°C on each of the food substrates for each day of development is presented with the day of spectral measuring.

Spectral Measuring	Day of development	ADD	Beef liver	Beef heart	Pork liver	Pork Heart
—	Day 0	0	Eggs	Eggs	Eggs	Eggs
—	Day 1	21.3	1st instar	1st instar	1st instar	1st instar
—	Day 2	42.8	2nd instar	2nd instar	2nd instar	2nd instar
—	Day 3	64.1	all 2nds	all 2nds	all 2nds	all 2nds
—	Day 4	84.8	3rd instar	3rd instar	3rd instar	3rd instar
—	Day 5	105.4	^1^Postf day1	Postf day1	3rd instar	3rd instar
Day 1	Day 6	126.1	Postf day2	Postf day2	Postf day1	Postf day1
Day 2	Day 7	146.8	Postf day3	Postf day3	Postf day2	Postf day2
Day 3	Day 8	167.4	Postf day4	Postf day4	Postf day3	Postf day3
Day 4	Day 9	187.8	Postf day5	Postf day5	Postf day4	Postf day4
Day 5	Day 10	208.3	^2^Pupa day1	Pupa day1	Postf day5	Postf day5
—	Day 11	228.9	Pupa day2	Pupa day2	Pupa day1	Pupa day1
—	Day 12	249.7	Pupa day3	Pupa day3	Pupa day2	Pupa day 2
—	Day 13	270.2	Pupa day4	Pupa day4	Pupa day3	Pupa day3
—	Day 14	291.6	Pupa day5	Pupa day5	Pupa day4	Pupa day4
—	Day 15	313.4	Pupa day6	Pupa day6	Pupa day5	Pupa day5
—	Day 16	333.4	Pupa day7	Pupa day7	Pupa day6	Pupa day6
—	Day 17	353.4	Pupa day8	Pupa day8	Pupa day7	Pupa day7
—	Day 18	373.3	Pupa day9	Pupa day9	Pupa day8	Pupa day8
—	Day 19	393.2	Pupa day10	Pupa day10	Pupa day9	Pupa day9
—	Day 20	413.1	Adults	Adults	Adults	Adults

^1^Postf = post feeding

^2^Pupa = intra-puparial period

Examinations of the spectral measurements of the post feeding larvae raised on each of the substrates were made in relation to beef liver, as beef liver is a substrate that is used regularly to rear blow flies in laboratory research [34].

**Fig 1:**
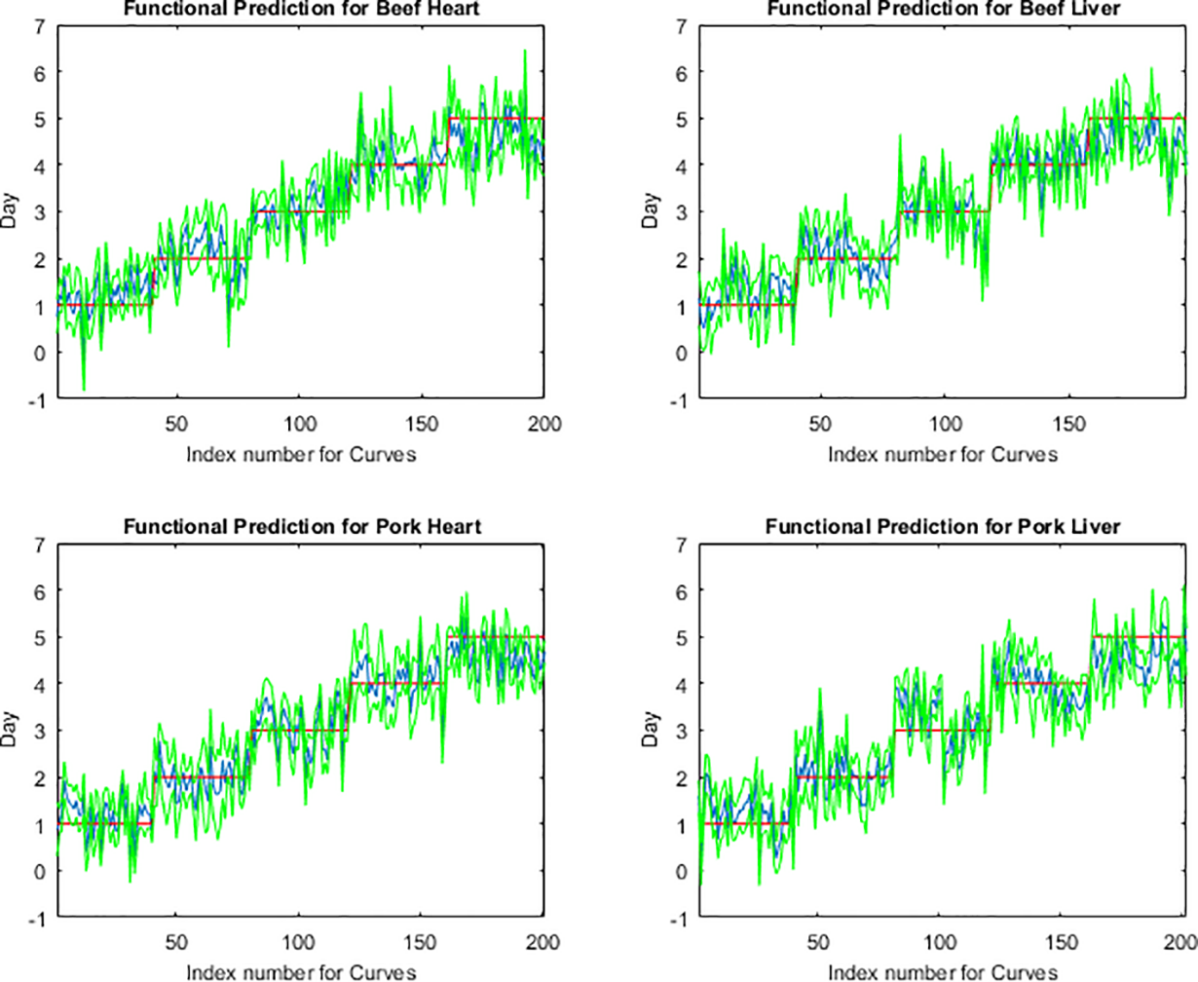
The functional regression prediction for day of development in the post feeding stage of *Lucilia sericata* raised at a mean temperature of 20.6°C on beef liver, pork liver, beef heart and pork heart for the anterior end spectral measurements with 95% prediction intervals (green lines are the upper and lower limits). The red line is the actual day of development in the post feeding stage and the blue is the predicted day based on the spectral measurements of each measured individual insect.

**Fig 2:**
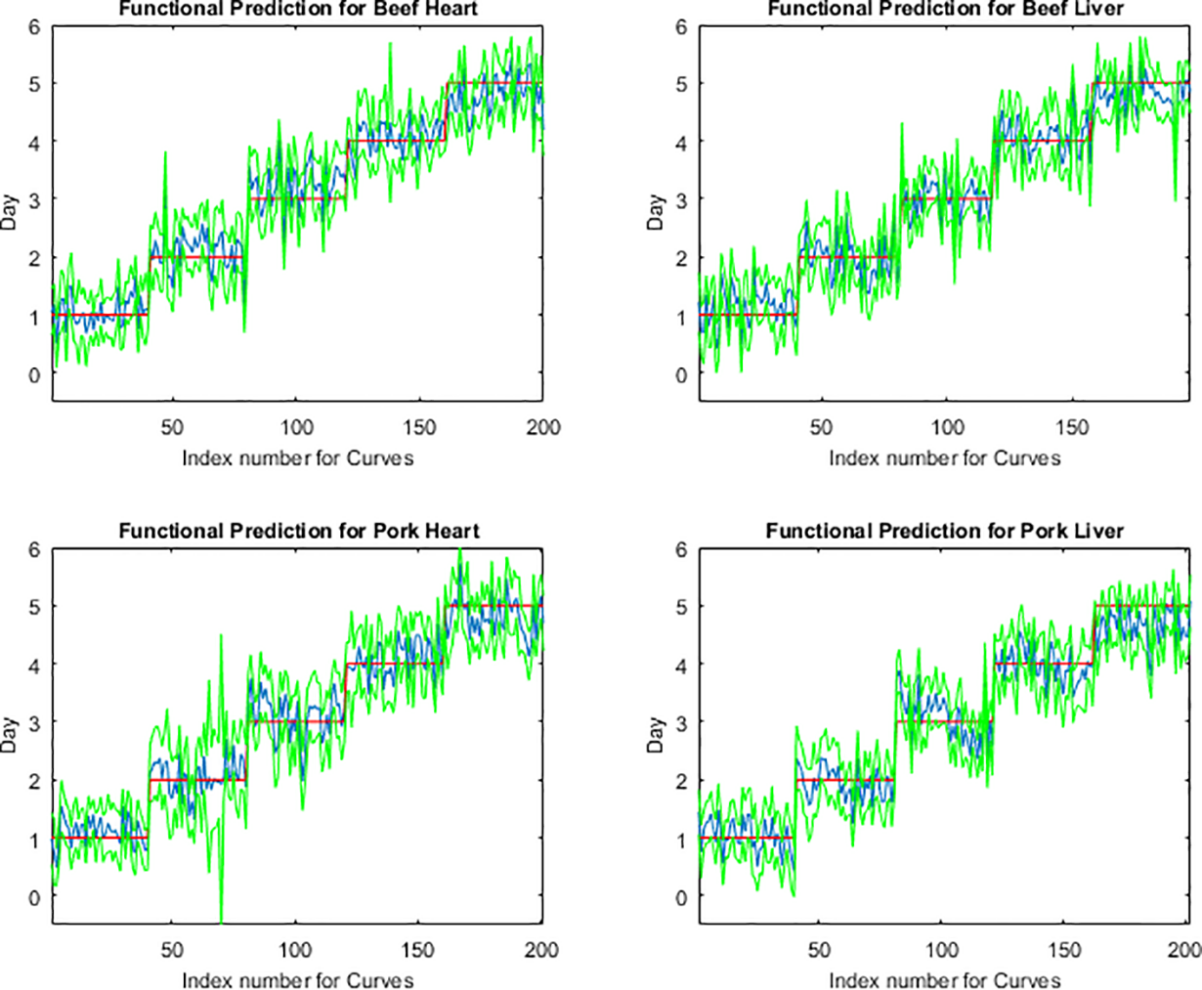
The functional regression prediction for day of development in the post feeding stage of *Lucilia sericata* raised at a mean temperature of 20.6°C on beef liver, pork liver, beef heart and pork heart for the midsection spectral measurements with 95% prediction intervals (green lines are the upper and lower limits). The red line is the actual day of development in the post feeding stage and the blue is the predicted day based on the spectral measurements of each measured individual insect.

**Fig 3:**
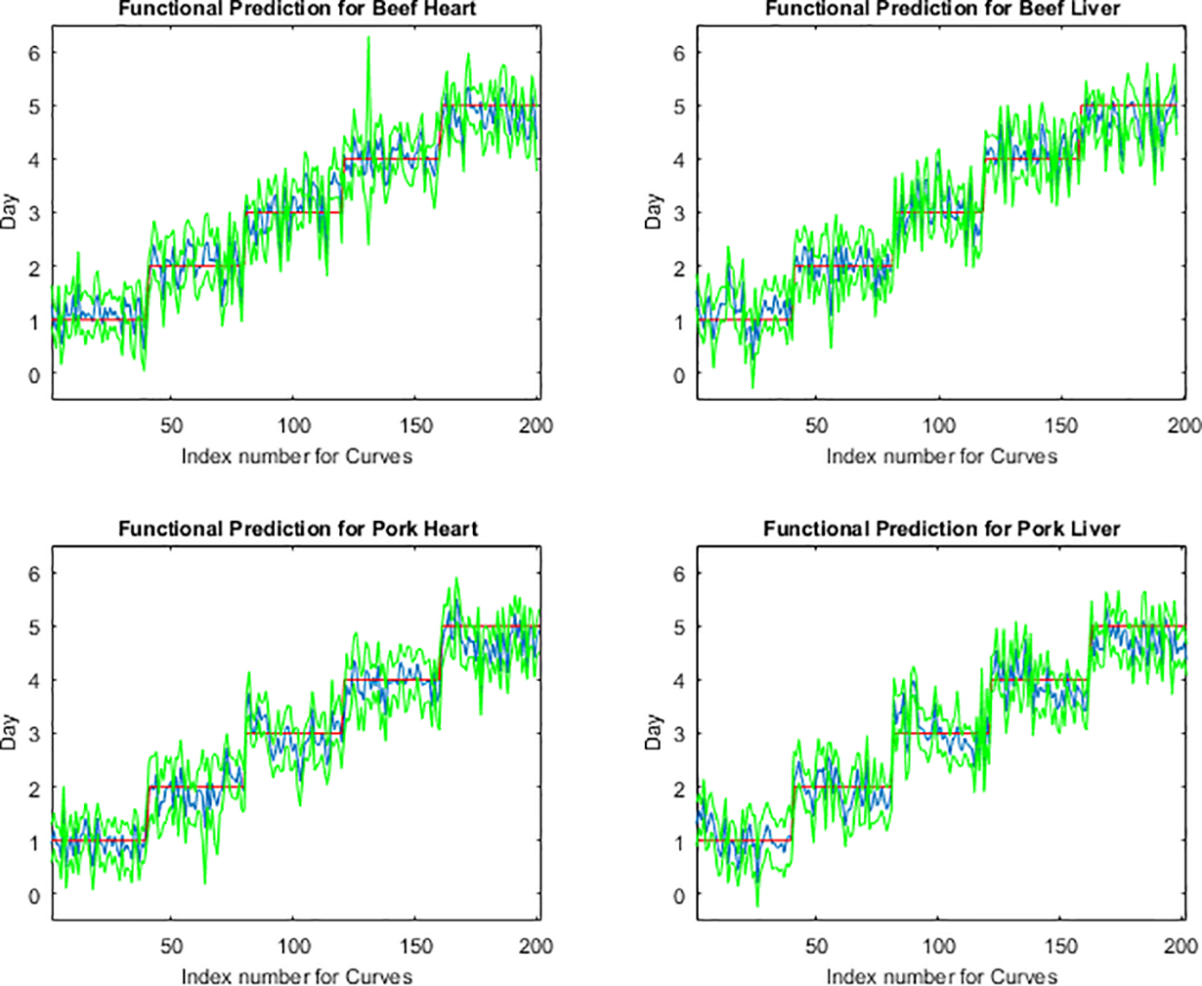
The functional regression prediction for day of development in the post feeding stage of *Lucilia sericata* raised at a mean temperature of 20.6°C on beef liver, pork liver, beef heart and pork heart for the posterior end spectral measurements with 95% prediction intervals (green lines are the upper and lower limits). The red line is the actual day of development in the post feeding stage and the blue is the predicted day based on the spectral measurements of each measured individual insect.

The functional regression model fits for the post feeding stage from measurements of the posterior end, anterior end and midsection are presented in Figs 1, 2 and 3, respectively. The measurements from the midsection and posterior end of the post feeding larvae outweigh the spectral measurements from the anterior end for predicting day within the post feeding stage. The actual day of post feeding development falls outside of the 95% prediction interval more often in the anterior measurements (Fig 1) than the midsection and posterior end measurements (Figs 2 & 3) in the functional regression plots. Also, most days of development are clearly distinguished from each of the other days in the post feeding stage in the midsection and posterior measurements.

As well as the functional regressions, the mean squared error (MSE) indicates that the functional prediction on the meat types have the highest error on anterior end measurements (Table 2). Therefore, it has the least accurate prediction capability compared with the midsection and the posterior end of the larvae. To confirm this, the overall percentage of true measurements falling outside of the 95% prediction interval for the anterior end, midsection and posterior end is 40.9%, 25.1% and 31.3%, respectively. The poor prediction capability of the anterior measurements is due to days one and five. This is evident when examining the anterior functional prediction plots in relation to the functional prediction plots of the midsection and posterior end. Many of the true values that tend to fall outside of the 95% prediction interval are from days one and five.

**Table 2:** The fixed effect models for the hyperspectral measurements of the anterior end, midsection and posterior end of post feeding *Lucilia sericata* raised on beef heart, (BH), beef liver (BL), pork heart (PH), and pork liver (PL).

Fixed Effect Models						
Region measured	Meat	Mean Squared Error (MSE)	# Outside	Total #	% of time true value falls outside interval estimate	% total outside interval for region
**Anterior end**	BH	0.2320	77	200	38.5%	
	BL	0.2630	87	197	44.2%	
	PH	0.1973	74	201	36.8%	
	PL	0.2767	89	202	44.1%	40.9%
**Midsection**	BH	0.1235	44	200	22.0%	
	BL	0.1375	57	197	28.9%	
	PH	0.1093	45	201	22.4%	
	PL	0.1272	55	202	27.2%	25.1%
**Posterior end**	BH	0.1159	58	200	29.0%	
	BL	0.1435	68	197	34.5%	
	PH	0.1108	52	201	25.9%	
	PL	0.1286	72	202	35.6%	31.3%
MSE subtotals	Anterior	0.9690				
	Midsection	0.4975				
	Posterior	0.4988				

In addition to the body region findings, the post feeding larvae that were raised on pork heart have the lowest MSE and the lowest number of times that the hyperspectral measurement falls outside of the 95% confidence interval for each body region. It is lowest for pork heart except with midsection measurements where with beef heart the percent of time that the true value falls outside of the 95% confidence interval is lower by a minimal difference of 0.4% than it is with post feeding larvae that were raised on pork heart.

**Fig 4:**
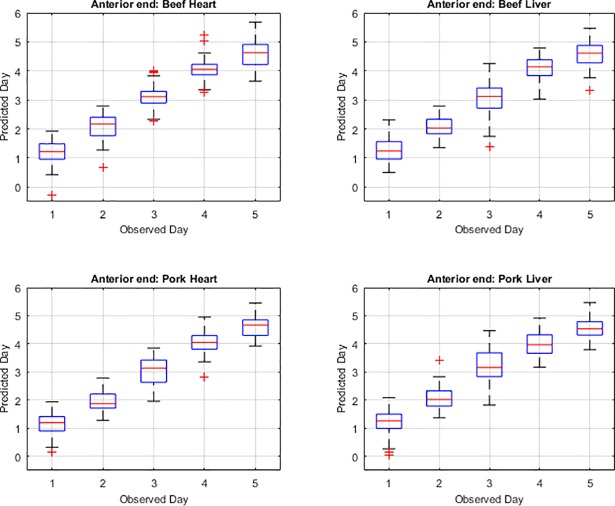
Box plots presenting the anterior end hyperspectral measurement prediction of day compared with the observed or actual day of *Lucilia sericata* post feeding larval development on beef heart and liver and pork heart and liver. The red line within the box represents the median and the box represents the upper and lower 50% of measurements. The boxplot whiskers extend up to a maximum of 1.5 times the interquartile range of the data. The observations beyond those points are shown as outliers and denoted by the “+” symbol.

**Fig 5:**
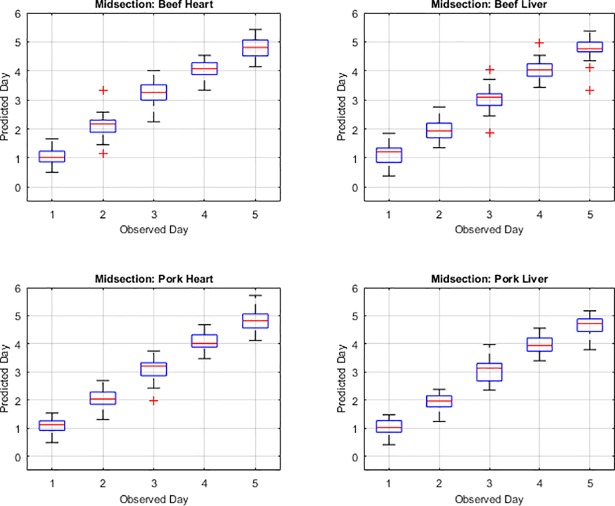
Box plots presenting the midsection hyperspectral measurement prediction of day compared with the observed or actual day of *Lucilia sericata* post feeding larval development on beef heart and liver and pork heart and liver. The red line within the box represents the median and the box represents the upper and lower 50% of measurements. The boxplot whiskers extend up to a maximum of 1.5 times the interquartile range of the data. The observations beyond those points are shown as outliers and denoted by the “+” symbol.

**Fig 6:**
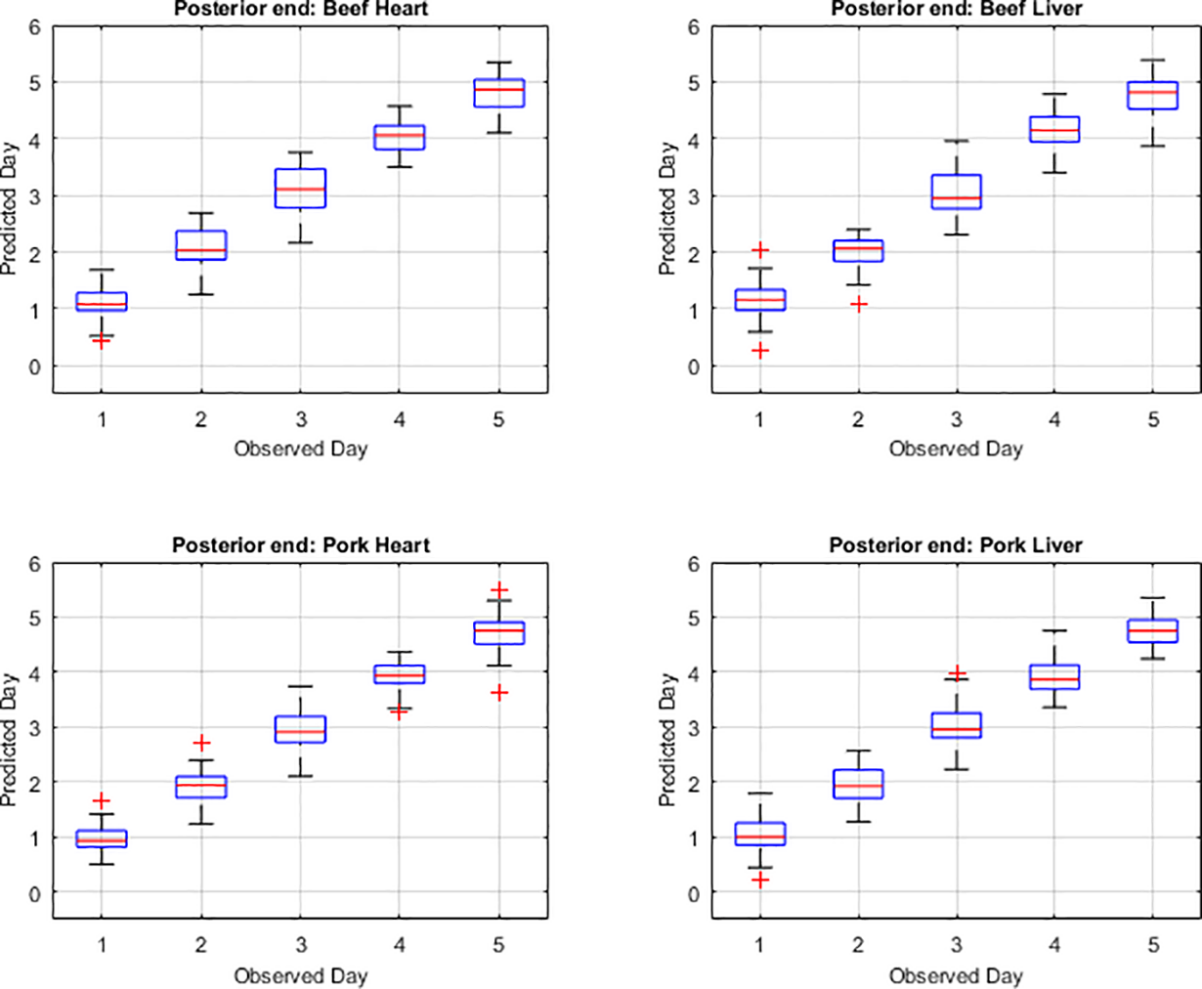
Box plots presenting the posterior end hyperspectral measurement prediction of day compared with the observed or actual day of *Lucilia sericata* post feeding larval development on beef heart and liver and pork heart and liver. The red line within the box represents the median and the box represents the upper and lower 50% of measurements. The boxplot whiskers extend up to a maximum of 1.5 times the interquartile range of the data. The observations beyond those points are shown as outliers and denoted by the “+” symbol.

In a comparison between the observed day and predicted day and the uncertainty associated with the predicted day for each of the measured body regions, it is evident that days one and day five provide the weakest prediction (Figs 4, 5 & 6). In the anterior end measurement plots of predicted versus observed day (Fig 4), days two, three and four predictions are similar to the observed or actual day and days one and five predictions are least like the observed day which is consistent with the mean squared error findings. In the midsection measurement plots of predicted versus observed day (Fig 5), in most days, the predicted day falls within the inter-quartile range and the median measurements predict the observed day. The predicted day based on midsection spectral measurements of post feeding larvae raised on beef heart for day three did not match the observed day within the interquartile range but did just outside in the lower whisker or 25^th^ percentile of measurements. For post feeding larvae raised on both beef and pork liver, the predicted day from the spectral measurements for day five falls outside of the interquartile in the upper whisker and so the observed day does not fall in the middle 50% of measurements. The median prediction based on posterior spectral measurements of the post feeding larvae that were raised on beef heart and liver and pork heart and liver was accurate for days one, two, three and four but was just outside in the upper whisker of the interquartile for day five (Fig 6). The predicted day falls closest to the observed day in the midsection and posterior measurement compared to the anterior measurements. Prediction of development day was most accurate based on the models that examined insects raised on pork heart.

**Fig 7:**
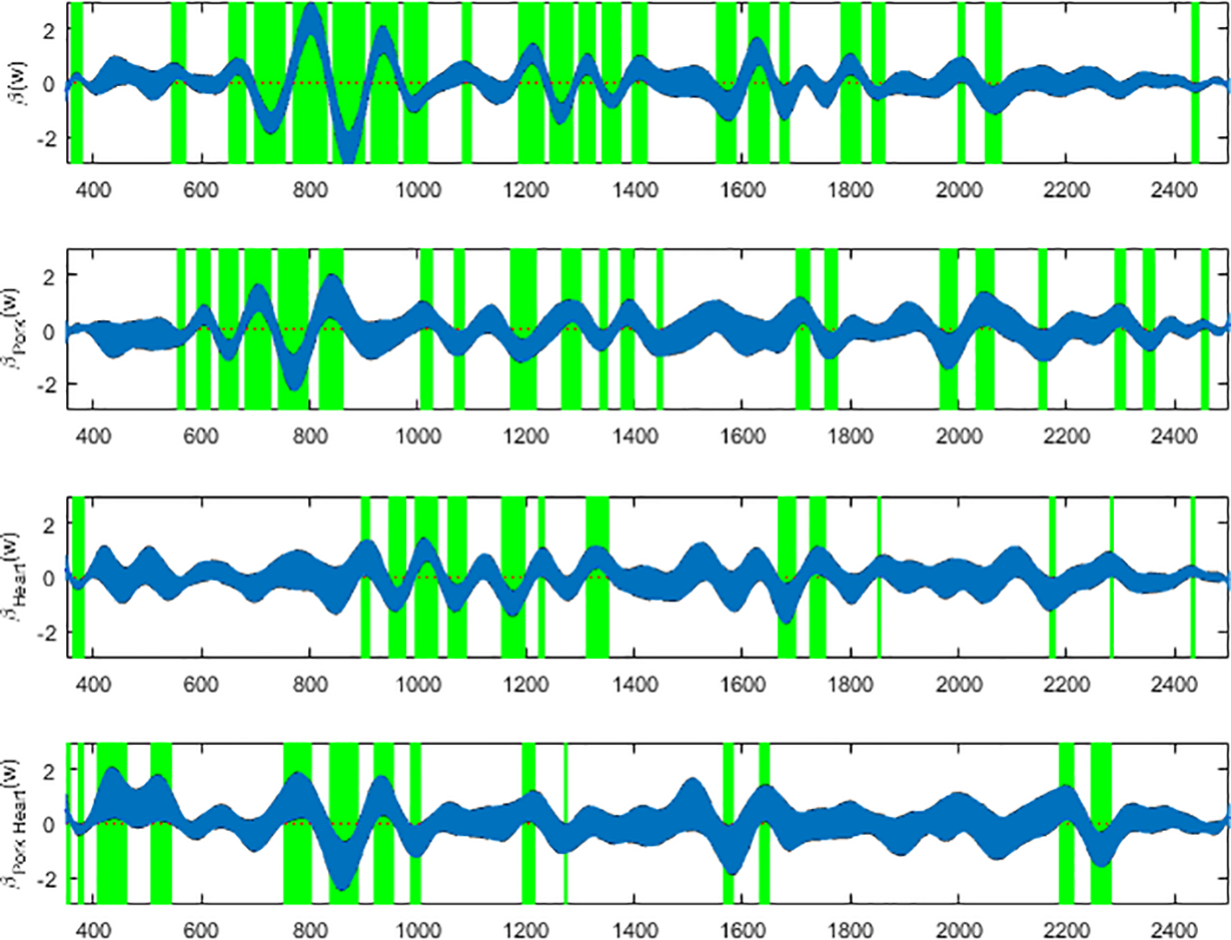
The *β(w)* coefficients (y-axis) and contributing wavelengths to the coefficients of the linear regression covariate model for the spectral measurements of the anterior end of post feeding *Lucilia sericata*. The blue area represents the 95% confidence interval and the green bands indicate wavelengths where *β(w)* coefficients are significant. *β(w)* is the contributing *β* coefficient for spectral measurements from insects raised on beef liver alone. *β*_*Pork*_*(w)* is the contributing *β* coefficient due to changing from beef to pork measurements regardless of organ. *β*_*Heart*_*(w)* is the contributing *β* coefficient due to changing from liver to heart measurements regardless of meat type. *β*_*Pork Heart*_*(w)* is the contributing *β* coefficient due to changing from beef liver to pork heart.

**Fig 8:**
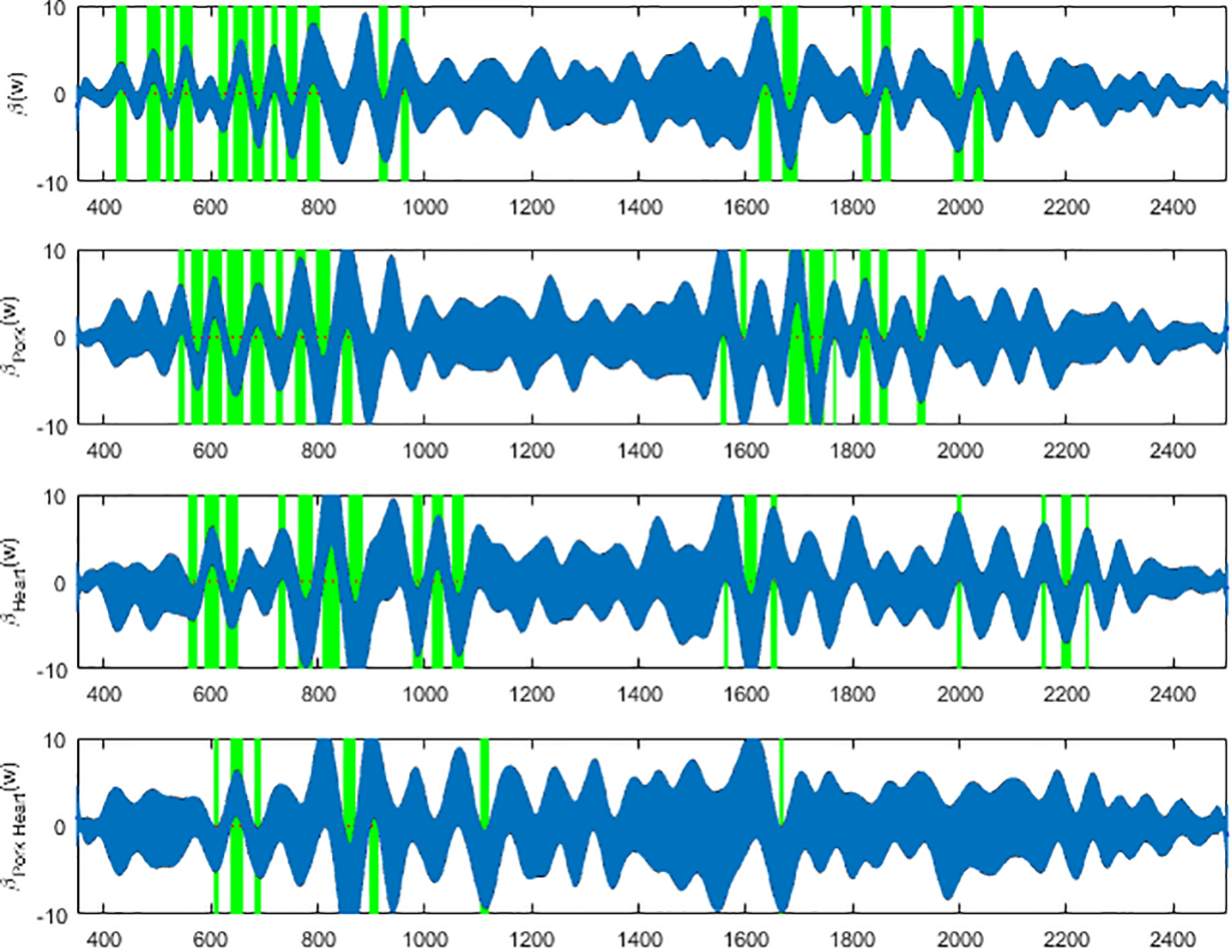
The *β(w)* coefficients (y-axis) and contributing wavelengths to the coefficients of the linear regression covariate model for the spectral measurements of the midsection of post feeding *Lucilia sericata*. The blue area represents the 95% confidence interval and the green bands indicate wavelengths where *β(w)* coefficients are significant. *β(w)* is the contributing *β* coefficient for spectral measurements from insects raised on beef liver alone. *β*_*Pork*_*(w)* is the contributing *β* coefficient due to changing from beef to pork measurements regardless of organ. *β*_*Heart*_*(w)* is the contributing *β* coefficient due to changing from liver to heart measurements regardless of meat type. *β*_*Pork Heart*_*(w)* is the contributing *β* coefficient due to changing from beef liver to pork heart.

**Fig 9:**
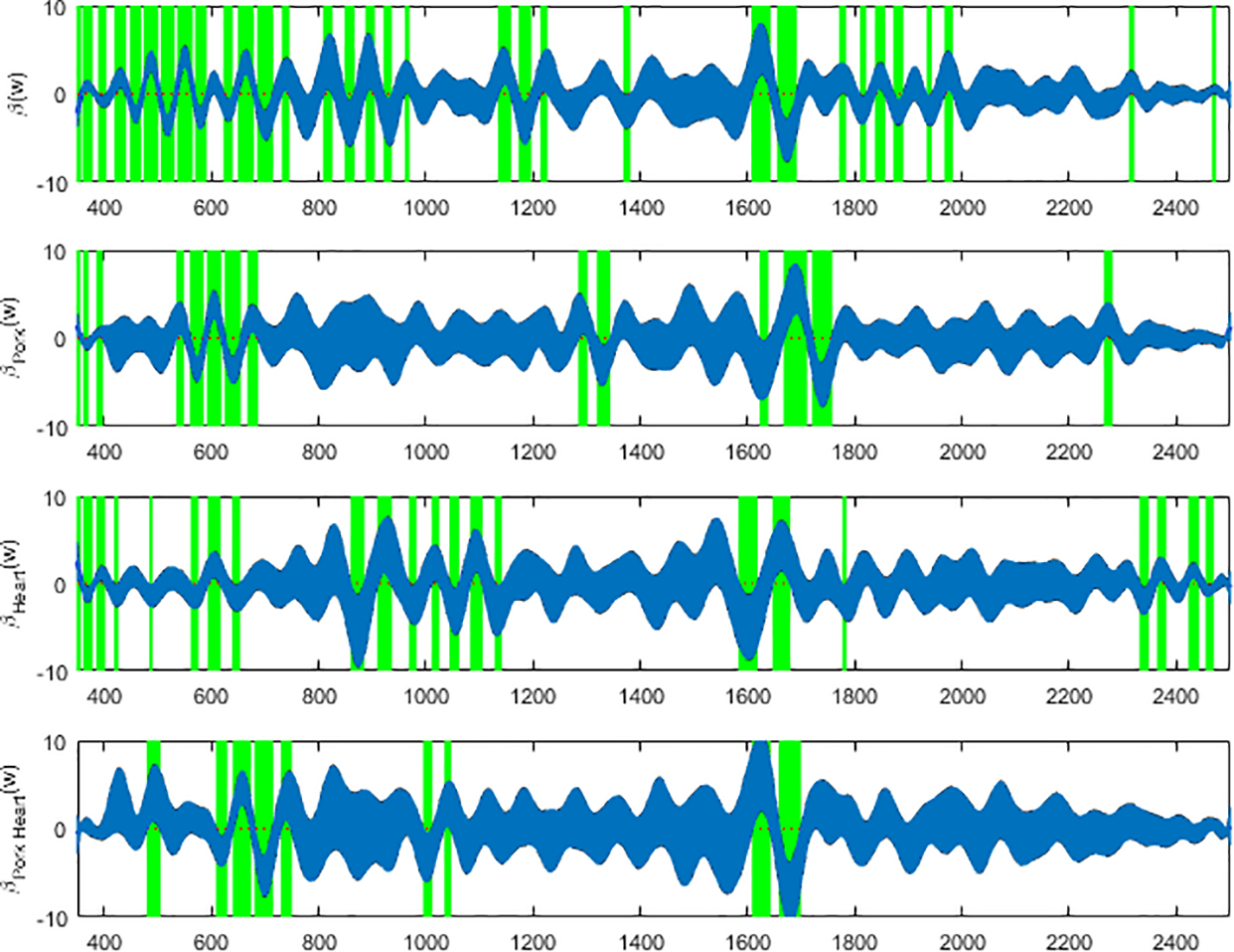
The *β(w)* coefficients (y-axis) and contributing wavelengths to the coefficients of the linear regression covariate model for the spectral measurements of the posterior end of post feeding *Lucilia sericata*. The blue area represents the 95% confidence interval and the green bands indicate wavelengths where *β(w)* coefficients are significant. *β(w)* is the contributing *β* coefficient for spectral measurements from insects raised on beef liver alone. *β*_*Pork*_*(w)* is the contributing *β* coefficient due to changing from beef to pork measurements regardless of organ. *β*_*Heart*_*(w)* is the contributing *β* coefficient due to changing from liver to heart measurements regardless of meat type. *β*_*Pork Heart*_*(w)* is the contributing *β* coefficient due to changing from beef liver to pork heart.

Based on the coefficient functions (Figs 7, 8 & 9), the ranges of wavelengths that are significant and contributing to the prediction for each meat type can be identified and were highlighted with green vertical bands. The green highlighted bands are the regions of wavelengths where the null hypothesis that there is no regression effect for predicting the day of development is rejected at the 5% significance level. Equivalently, these blue bands in Figs 7–9 show the confidence intervals for the regression coefficient effects across wavelengths. The regression effect is particularly evident at wavelengths 350-800nm except for the anterior measurements where the greatest contributions appear to fall between 900 and 1350nm. Each of the regression coefficient functions have significant non-zero effect regions and therefore, the null hypotheses for *L*. *sericata* raised on all the meat types were rejected at p≤0.05 for at least some wavelength bands. The contributing wavelengths for each of the coefficient functions differ, in some cases only slightly, from each other when examining the effect of the organ type and meat type and the interaction effect of different organ and different meat type with pork heart.

## Discussion

*Lucilia sericata* raised on beef liver and heart increased in size visibly faster than those raised on pork liver and heart. They fed for one day less on beef organs than they did on pork organs. The larvae feeding on the pork organs were noticeably much smaller in size but increased in size with the extra day of feeding. These findings were very different from previous findings, which indicate that *L*. *sericata* grew faster on pork lung, liver and heart than on the same beef tissues [41]. Differences between the findings for the same species could be a result of geographically separate populations, as it is probable that the earlier research was performed on *L*. *sericata* trapped in the United Kingdom [42, 43]. Genetic and phenotypic differences have been found in *L*. *sericata* from environmentally separate populations and ecological differences may be a contributor [44]. A temperature and size relationship between strains was found in the studied populations [44].

Interestingly, adult *L*. *sericata* reared on beef or pork began emerging on the same day, and so the *L*. *sericata* raised on pork spent one less day in the intra-puparial period. The nutritional value of pork heart and liver does not explain the need for the extra day of feeding in comparison with the beef organs (Table 3) but fatty acids may. Fatty acids increase the oily consistency of the meat substrate [45] and the beef heart was noticeably oilier than the pork heart. The oily consistency of the beef and pork liver was not detectable because of the moist surface consistency of liver. Since beef animals primarily have a grass diet, their vitamin E intake is higher thereby increasing their poly unsaturated fatty acid (PUFA) levels at slaughter to higher than that of pork [45]. Without Vitamin E in a ruminants’ diet, however, oxidation of the fatty acids is faster than that of porcine following slaughter [45]. Vitamin E slows the oxidation of PUFAs and causes an oily consistency [45], which may have made it easier for the third instar larvae to break the surface of the beef substrates when feeding compared with the larvae feeding on the pork substrates accounting for the extra day of feeding. There was no delay with the earlier larval stages feeding on pork and this is probably because there was enough liquid protein in those first feeding days for the less developed mouth parts of first and second instar larvae.

**Table 3:** Nutritional value per 100g of meat substrate.

	Beef liver	Beef heart	Pork liver	Pork heart
Calories/ fat cal	135/32.7	112/35.5	134/32.9	118/39.3
Fat total/sat (g)	3.6/1.2	3.9/1.4	3.6/1.2	4.4/1.2
Cholesterol(mg)	275	124	301	131
Carbohydrates (g)	3.9	0.1	2.5	1.3
Protein (g)	20.4	17.7	21.4	17.3
Water content (g)	70.8	77.1	71.1	76.2

(http://nutritiondata.self.com/ accessed Jun04 2017)

One of the four replicates for each of the pork liver and pork heart took more time (an extra day for pork heart and an extra two days for pork liver) for the adults to begin emerging. This was probably due to much greater mortality in these replicates as higher mortality was observed in these slower developing replicates.

The anterior measurement median predictions are consistent with the observed day for days two, three and four but are not for days one and five of the post feeding stage. These results support the findings of the higher mean squared error for the anterior measurements. The median prediction based on midsection spectral measurements of post feeding larvae that were raised on pork heart according to the box plots was most consistent with the observed day as compared with development on the other meat types and organs. This is consistent with the mean squared error findings. The lowest means squared error was observed with development on pork heart and spectral measurements of the midsection. The accurate median prediction for most days but day five from the posterior measurements is consistent with the low mean squared errors from the posterior end measurements of post feeding larvae raised on all the meat types. Posterior end measurements are usually superior to midsection and anterior end measurements when examining post feeding larvae [19, 20]. The weaker Day 5 prediction from the posterior end measurements would, however, explain the higher mean squared error subtotal for the posterior end measurements than the midsection measurements.

Based on the coefficient functions, Figs 7, 8 & 9, there are fewer significant wavelengths for pork heart, particularly in the midsection and posterior measurements, this can potentially explain the lower MSE for pork heart compared with the other meat types (Table 2). Surprisingly the MSE is very slightly lower in the midsection measurements than the posterior end measurements for predicting day of development. Previous findings have found that prediction based on posterior measurements has outweighed those of anterior and midsection measurements for *P*. *terraenovae* [20] and *L*. *sericata* [19] when raised on veal liver and beef liver, respectively. The slightly lower MSE subtotal for midsection is probably a result of the lower pork heart MSE and the lower percent of times that the true value for beef heart fell outside of the 95% confidence interval (Table 2). The true value fell outside of the 95% confidence interval only 0.4% fewer times for the post feeding larvae that were raised on beef heart than those raised on pork heart. From an overall perspective, the majority of the wavelengths at which measurements were taken do not contribute to the prediction as their functional coefficients are not significant and focus can remain on those wavelengths identified in Figs 7, 8 & 9.

The spectral measurements from the midsection and posterior end of the *L*. *sericata* larvae were found to be superior for predicting the day within the post feeding stage as compared with anterior measurements. This is probably a result of the ectodermal oenocytes which produce cuticular hydrocarbons [46]. They are often located in the abdomen of the larvae in close proximity to the spiracles but are species and stage dependent [47]. The cuticular hydrocarbons are then transported by lipophorin in the haemolymph to the remaining cuticle and fat body [47, 48]. The oenocytes have been found to grow and form new variations with each moult [48, 49], and so it is very probable that the change in oenocytes may result in changes to the cuticular hydrocarbons.

The anterior end of the larvae was found to be particularly poor for predicting age of larvae and there may have been several causes for this. First, the anterior end was a smaller target and the larvae had a tendency to move their anterior regions away from the fiber optic probe when attempting to position them and hold them still for long enough to complete a measurement. Second, the cuticular hydrocarbons may potentially not be as abundant or were lacking precision in delivery to that region since it is farthest from the oenocytes and the hydrocarbons require transport to this region. Third, feeding has stopped upon entering the post feeding stage and so there may no longer be a release of digestive enzymes potentially laced with bacteria on the anterior end of the insect surface [15]. Day five prediction was probably least convincing because the insects were transitioning from the post feeding stage to the intra-puparial period and so was reducing its transport of cuticular hydrocarbons to the insect surface in preparation for apolysis. This would reduce the changes to the insect cuticle and make it more difficult to distinguish from the previous day as was seen in Figs 1, 2 & 3 where the blue prediction line somewhat blurs between the last two days. This is much more evident in Fig 1, the anterior end measurements.

The experiments showed that the food substrate on which insects are raised does have a minimal effect on the day of development prediction from spectral measurements. The functional regressions from each body region indicated that, when examining the effect of spectral measurements from insects raised on pork compared with those on beef, there is an effect on predicting day within the post feeding stage. Similarly, when examining the effect of *L*. *sericata* spectral measurements raised on heart compared with those raised on liver, there was also an effect. Day predictions within the post feeding stage were also affected when examining the interactional effect of both organ and meat type, pork heart in reference to beef liver.

It is most probable that differences in the cuticular hydrocarbons are due to differences in the food substrates since diet has been shown to affect *Drosophila* spp. and ants [50–53]. There is a strong possibility that the fatty acids in the food substrates were impacting the cuticular hydrocarbon profile since this has been reported to occur in the herbivorous mustard leaf beetle, *Phaedon cochleariae* (F.). The mustard leaf beetle was fed artificial diets of fatty acids and this resulted in changes to the straight chain and methyl-branched cuticular hydrocarbons [54].

Based on the coefficient functions (Figs 7, 8 & 9), the significantly nonzero portions of the *β*_*Pork Heart*_*(w)* functions that are contributing to the interactional effect are not as numerous as the contributing *β* *(w)* coefficients in the beef liver alone model. Also, for the meat and organ type effect, there are missing and extra contributing *β(w)* coefficients that are not contributing to the beef liver alone model. Hence the significance of different wavelength regions within all of the coefficient functions across all of the body regions shows that there are additional wavelengths of spectral measurements contributing to differentiating the model during changes in meat type and organ choice. The *β* coefficients indicate whether or not a significant relationship exists between wavelength and spectral reflectance for the measured insects and also indicates at which wavelengths a significant relationship exists. Although day predictions are accurate, the differences in *β* coefficients and therefore; different contributing wavelengths indicate why care must be taken when using spectral measurements to age larvae raised on different food substances. This is particularly important when applying findings from different food substrates to casework.
